# Progress in Genito-Urinary Surgery

**Published:** 1903-03-14

**Authors:** 


*(To be Continued.)*

### Progress in Genito-Urinary Surgery.

**The Pathology and Ætiology of Senile Enlargement of the Prostate.**—L. R. G. Crandon, of Boston,[1] has contributed an interesting paper on the pathogenesis and pathological anatomy of enlarged prostate. He states that he undertook the work described, largely with the view of investigating the conclusions of Ciechanowski, to which we refer below. Of men with the symptoms of prostatism, *i.e.* increased frequency of micturition, incomplete emptying of the bladder, with residual urine, or distension with dribbling, it has been estimated that only 15 per cent. have enlarged prostates. Crandon first records his careful investigations with reference to the condition of the bladder wall in vesical insufficiency. In half the subjects over forty-five years of age, a sagittal section through the bladder and urethra shows a distinct lip rising above the level of the urethral floor at its origin and then dropping sharply behind to form the anterior wall of a bladder-pouch. The prostrate in the presence of this lip may or may not be enlarged. The pouch is a prominent factor in prostatism. The writer concludes that though the cause of bladder insufficiency may alone be structural changes in the bladder-wall, including the atrophy of muscle and the new growth of connective tissue infiltrating the muscles, yet the most important factor is probably obstruction at the beginning of the urethra. The obstruction may be due simply to the lip mentioned above or to true enlargement of the whole or part of the prostate. The most marked cases of prostatism are those in which atrophy of bladder-muscles, connective-tissue infiltration, enlarged prostate, and chronic inflammation are all present. The author next records his investigations into the fine and gross structure of the enlarged prostate. In his conclusions he reaffirms the statements of Ciechanowski. According to these views the common starting point of the enlargement, and also of certain forms of atrophy of the prostate is to be sought in the production of connective tissue in the stroma of the gland. If this tissue is produced in the central parts of the gland, near the exits of the glandular ducts, the glandular follicles dilate, proliferate, desquamate, etc., and the enlargement of the prostate is almost exclusively due to these changes in the glandular elements. If the connective tissue is produced principally in the periphery of the gland, near the blunt ends of the tubules, then there is atrophy of these and of the entire prostate. Atrophy and enlargement of the prostate are therefore probably etiologically similar. Summing up the conclusions as to the cause of enlarged prostate the author states his belief that the changes mentioned above are due to infection, usually gonorrhœal, or to infection aggravating a senile degenerative process, and that neoplasms, fibro-myomata and adenoma, though they occur, are rare.

**The Operative Treatment of Enlarged Prostate.**—At the Manchester meeting of the British Medical Association, P. J. Freyer [2] again discussed his operation of total extirpation of the prostate, and showed all the prostates he had removed by this method, 21 in number. The shape of these prostates differed much, but they were all more or less conical with the base directed towards the triangular ligament and the apex into the bladder. This is no doubt due to the deficiency of space beneath the pubic arch outside the bladder forcing the gland as its size increases to pass into the bladder between the posterior margins of the recto-vesical fascial sheath, and many of the specimens showed a circular groove caused by the grip of this unyielding fibrous and muscular tissue. Freyer classified his specimens in three groups: (1) Those in which the lateral lobes had separated along their superior commissures only during removal; (2) Those in which the lobes had separated along both commissures and the two halves had been separately removed; (3) Those in which the lobes had not separated at either commissure, and in which, therefore, during removal, the urethra and ejaculatory ducts had been torn across. In each case the prostate was covered by a thin, strong fibrous membrane, the true capsule of the prostate as described by Sir Henry Thompson. In operating, the mucous membrane over the intra-vesical swelling is scraped through with the sharpened finger nail. The prostate in this part has often already burst through its sheath, and is merely covered by mucous membrane, and on scraping through this the capsule proper is at once reached. The finger then separates the gland, passing between its sheath and capsule proper, and it is important not to penetrate the latter or the finger will flounder about and only enucleate isolated adenomatous tumours instead of the whole organ. In all save two or three of Freyer's cases complete catheter life had been entered upon. All the patients were broken in health, some almost moribund. In 19 an absolute cure had resulted. Parker Syms,[3] also at the Manchester meeting, described a special method of performing perineal prostatectomy. He stated that this operation provided a safe and scientific method of radically curing the disease under consideration. In suprapubic prostatectomy the bladder was mutilated in two places, and there was left by the operation a lacerated bladder floor and a more or less large blind pouch beneath the bladder from which the prostate had been removed, and convalescence was prolonged, distressing and dangerous. In the operation described a median perineal incision is made on to a staff, and the full length of the membranous urethra divided. The incision is deepened till the apex of the prostate is reached. The prostatic urethra is dilated with a divulsor and the author's rubber retractor introduced into the bladder. The rubber bag at the bladder end of this instrument is then distended with water, and when the stem is pulled upon the prostate is brought well within reach, so that the operator can enucleate its lobes with his index finger. The fibrous sheath of the gland is incised with scissors, the line of cleavage found with the finger, and enucleation performed. No mucous membrane is removed, though the prostatic urethra is usually divided. After the operation a perineal drainage tube is inserted and the wound packed behind it. The tube is removed on the fifth or sixth day and then the patient is allowed to sit up and walk about. The author had operated on 21 cases, and had had no deaths, either immediate or remote. The patients subsequently had been able to hold their urine from two to four hours during the day, and had been cured of their residual urine. The author was convinced that the perineal operations were the best and safest for this disease. L. Rydigier [4] has also written recommending perineal operations, and states that Albarran has successfully performed the operation on a large number of cases. Rydigier considers it very important not to wound the urethra, and for that reason now advises that intracapsular resection of the prostate should be performed rather than enucleation of the gland, together with its capsule. He usually makes a median incision and exposes the posterior surface of the prostate, and then in turn on each side incises the capsule and proceeds to shell out the corresponding lobe. Usually this is easy, but in some cases the capsule is thin and adherent and easily torn. At a certain distance from the urethra, in which a thick catheter has been placed, a clamp forceps is applied to the spurious pedicle which has been formed. The instrument is placed parallel to the urethra, and the corresponding lobe then resected. The writer believes the functional results of this operation will be quite as good as those of total enucleation.

[1] Annals of Surgery, Dec., 1902, p. 813. [2] Brit. Med. Jour., Nov. 8, 1902, p. 1492. [3] Ibid., p. 1495. [4] Centralb. für Chir., Oct. 11, 1902, p. 1057.

